# 3D Interconnected Boron Nitride Networks in Epoxy Composites via Coalescence Behavior of SAC305 Solder Alloy as a Bridging Material for Enhanced Thermal Conductivity

**DOI:** 10.3390/polym12091954

**Published:** 2020-08-28

**Authors:** Youjin Kim, Jooheon Kim

**Affiliations:** School of Chemical Engineering & Materials Science, Chung-Ang University, Seoul 156–756, Korea; becon272727@gmail.com

**Keywords:** composites, epoxy, boron nitride, SAC305, thermal conductivity, 3D structure, coalescence

## Abstract

In this study, hybrid fillers of spherically shaped aggregated boron nitride (a-BN) attached with SAC305, were fabricated via simple stirring and the vacuum filtration method. a-BN was used as the primary conductive filler incorporated with epoxy resin, and these fillers were interconnected each other via the coalescence behavior of SAC305 during the thermal curing process. Based on controlled a-BN content (1 g) on 3 g of epoxy, the thermal conductivity of the composite filled with hybrid filler (a-BN:SAC305 = 1:0.5) reached 0.95 W/mK (33 wt%) due to the construction of the 3D filler network, whereas that of composite filled with raw a-BN was only 0.60 W/mK (25 wt%). The thermal conductivity of unfilled epoxy was 0.19 W/mK.

## 1. Introduction

High thermal conductive polymer composites have generated increasing interest because of the rising demand for thermal interface material (TIM) for efficiently dealing with overheating of electronic devices [1,2,3]. Polymers are widely used as TIMs due to their easy processability, low price, and low weight, but they have low thermal conductivity because of the complex morphology of polymer chains [4]. Fillers with high intrinsic thermal conductivity, such as ceramic, carbon materials, and metal oxides, are incorporated into a polymer matrix to address this problem [5,6,7]. The thermal conductivity of composites can be increased with the addition of filler, but a high concentration of filler would cause a loss of polymeric properties. Therefore, it is essential to improve the thermal conductivity of the composites by adding less filler to preserve polymeric properties.

Heat is carried by acoustic phonons in non-metallic fillers [8]. For polymer composites with thermally conductive fillers, phonons scatter at the filler–matrix interface, resulting in a limited increment of thermal conductivity. Moreover, the uniform dispersion of nanofillers would introduce more interfaces, causing more phonon scattering. The formation of filler agglomerate is sometimes more beneficial for heat transfer, because a filler–filler interface has fewer phonon mismatches than a filler-matrix interface. However, these fillers also have a disadvantage because of the large mean interparticle distances inside the matrix compared to nanofillers. The optimal point should be identified from both perspectives.

Recently, constructing a three-dimensional (3D) thermal path in a polymer matrix to enhance thermal conductivity has become an effective strategy [9]. Teng’s group reported the synthesis of a 3D boron nitride nanosheets (BNNS) network using the ice-templating method and connected these fillers with silver nanowire (AgNW) as “thermally conductive bridges” [10]. Xie’s group reported the construction of a 3D BNNS network with the coalescence behavior of AgNPs attached to the BNNS surface via the sintering process [11]. Wang’s group also constructed 3D filler networks of Al_2_O_3_ for high thermal and mechanical properties [12]. 3D networks can also be achieved using foam-like materials as thermally conductive paths [13,14,15,16]. These studies highlight the possibility of achieving high thermal conductivity of polymer composites with small filler fractions maintaining polymeric properties. However, the bridging materials used in these studies also have high thermal conductivity. Therefore, enhanced thermal properties result from not only the construction of 3D thermal paths but also the synergistic effect of the high intrinsic thermal conductivity of the bridging fillers.

In this study, we investigated the role of the bridging material in enhancing the thermal properties of composites using the coalescence behavior of SAC305 (Sn3.0Ag0.5Cu). SAC305, commonly used as solder powder, has a low thermal conductivity of ~59 W/mK compared to other conductive fillers, so the sole effect of bridging fillers on the increase in thermal conductivity can be investigated. The spherical shaped aggregated boron nitride (a-BN) particles were introduced as main conductive filler, and diglycidyl ether of bisphenol A (DGEBA) epoxy was used as the matrix. SAC305 particles were attached on the surface of a-BN by van der Waals attraction force, and various ratios of hybrid fillers were prepared (1:1, 1:0.5, and 1:0.2). The hybrid fillers were introduced into the epoxy resin solution mixing at 120 °C. The resulting slurry was cured at a melting point (mp) of SAC305 (220 °C) to connect a-BN fillers, which can reduce thermal resistance between the filler-matrix interface. The through-plane thermal conductivity of composites cured at the mp of SAC305 and below the mp was measured and compared to show the effect of coalescence behavior. Due to low mp of SAC305, the curing process and, simultaneously, connecting fillers to construct a 3D thermal transport path can be achieved without the thermal degradation of the epoxy matrix.

## 2. Experimental

### 2.1. Materials

a-BN (70 μm) particles were obtained from Saint-Gobain (Courbevoie, France). Solder powders (Sn/3.0Ag/0.5Cu, 2–11 μm, SAC305) were purchased from DUKSAN Hi-Metal Co., Ltd (Ulsan, Korea) Diglycidyl ether of bisphenol-A (DGEBA) was obtained by Kukdo Chemical Co. (Seoul, Korea). Finally, 4,4’-Diaminodiphenylmethane (DDM) was acquired from Sigma-Aldrich (St. Louise, MO, USA).

### 2.2. Preparation of a-BN/SAC305 Hybrid Filler

a-BN powders (2 g) were dispersed in 500 mL of water-ethanol mixed solvents (7:3 by volume) followed by the addition of SAC305 with various ratios (a-BN:SAC305 = 1:1, 1:0.5, 1:0.2) in each experiment. The resultant dispersions were stirred for 3 h at room temperature and vacuum filtrated. a-BN/SAC305 hybrid fillers were obtained after drying in a convection oven at 50 °C overnight.

### 2.3. Fabrication of Epoxy Composite Filled with a-BN/SAC305 Hybrid Filler

**Scheme 1:**
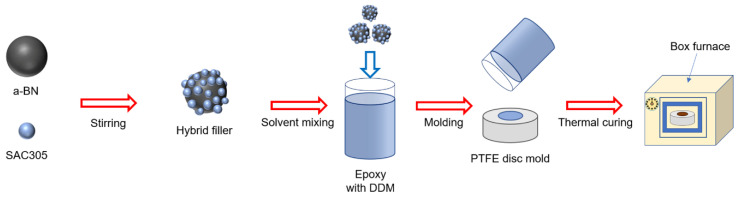
Fabrication procedure of epoxy composite filled with a-BN/SAC305 hybrid filler.

a-BN/SAC305 was dispersed in acetone and then added to DGEBA (2.14 g) to produce various filler weight fractions. The amount of filler and wt fraction in each experiment are denoted in Table 1. The resulting mixture was stirred at 120 °C until the acetone evaporated, followed by the addition of DDM (0.86 g) as a curing agent. After the DDM melted, the a-BN/SAC305/epoxy slurry was then poured into the disc-shaped mold (2 mm thickness, 25.4 mm diameter) and placed in a vacuum oven for 20 min at 50 °C to remove the bubbles inside the mixture. This mixture was then cured at 220 °C (higher than mp of SAC305) for 10 min, followed by post-curing at 180 °C for 20 min, to enable the SAC305 attached on the a-BN surface to coalesce with each other. Scheme 1 shows the experimental process.

### 2.4. Characterization

The morphology of the a-BN/SAC305 and the fractured surface of a-BN/SAC305/epoxy composites were obtained using a field-emission scanning electron microscope (FE-SEM, Sigma, Carl Zeiss, Oberkochen, Germany) and field-emission transmission electron microscope (FE-TEM, JEM-F200, JEOL, Akishima, Japan). A sample for the TEM image of the fractured surface of the composite was prepared using cryo-ultramicrotomy (PowerTome PC, RMC Boeckeler, Tucson, AZ, USA). Energy dispersive spectroscopy (EDS) was performed to detect the elemental distribution of a sample using an EDS detector equipped with FE-TEM. X-ray diffraction (XRD, New D8-Advance, Bruker-AXS, Billerica, MA, USA) analysis was performed with a 2θ range of 10–80°. The melting behavior of SAC305 during the curing process was detected using differential scanning calorimetry (DSC, DSC-7 system, Perkin-Elmer, Waltham, MA, USA). The thermal oxidation of BN/SAC305 particles was examined by thermogravimetric analysis (TGA, TGA-2050, TA-instrument, New Castle, DE, USA) at a heating rate of 10 °C /min in a range of room temperature (RT) to 800 °C in an air atmosphere. The thermal property of composites was investigated by measuring thermal diffusivity using laser flash analysis (LFA, Nanoflash LFA447, Netzsch Instruments Co., Selb, Germany). Thermal conductivity was calculated by multiplying diffusivity with density and specific heat capacity.

## 3. Results and Discussion

**Figure 1:**
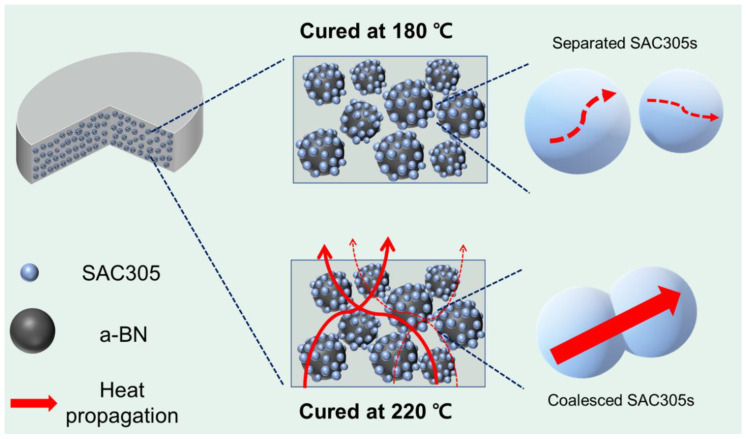
Schematic of fractured surface of the composites cured at 180 °C and 220 °C.

We constructed a 3D bridged structure of a-BN with the coalescence behavior of SAC305 particles to produce thermally-conductive epoxy composites (Figure 1). The a-BN/SAC305/epoxy composites were produced with a one-pot fabrication curing process and coalescence at 220 °C for 10 min, followed by post-curing at 180 °C for 20 min. SAC305, which is widely used as a solder powder, has an mp of 217–219 °C [17,18]. Another a-BN/SAC305/epoxy composite was fabricated with a curing temperature of 180 °C (lower than the mp of SAC305) for 30 min to compare the results to that of the thermal property of the sample without the coalescence effect. As depicted in Table 1, epoxy composites filled with a-BN/SAC305 hybrid filler (1:1 wt ratio) loadings of 10 wt%, 20 wt%, 30 wt%, and 40 wt% were prepared. We also fabricated epoxy composites with different ratios of hybrid fillers (1:0.5, 1:0.2); their BN content in 3 g of epoxy was set based on 1:1 wt ratio-hybrid fillers.

**Figure 2:**
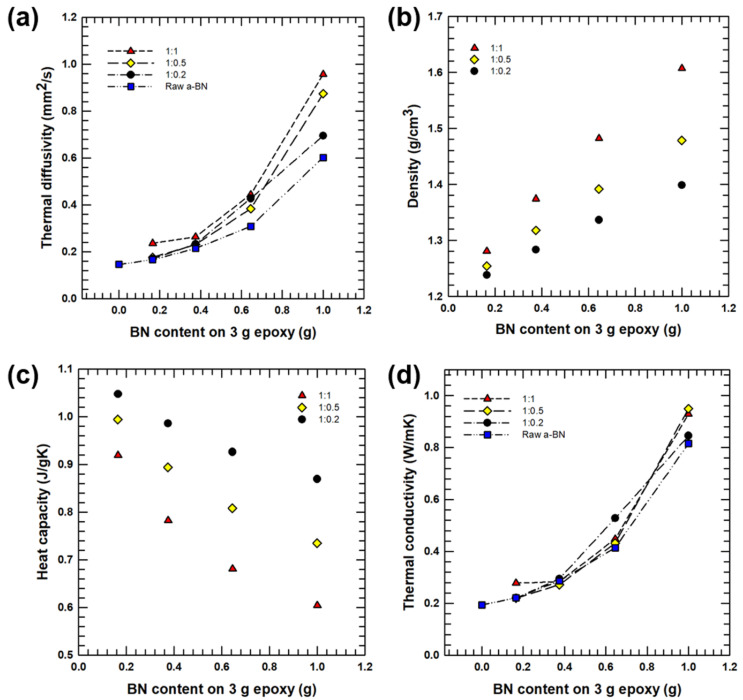
(**a**) Thermal diffusivity, (**d**) thermal conductivity of the composites with raw a-BN, and various ratio of the hybrid fillers. (**b**) Density, and (**c**) specific heat of the composites with different ratios of the hybrid fillers.

The thermal property of a-BN/SAC305/epoxy composites was investigated by measuring the thermal diffusivity of the composites (Figure 2a). Regardless of the type of filler, all composites exhibited steep increases when the BN content on 3 g of epoxy was 1 g. As depicted in Figure S1, the a-BN particles sink to the bottom of the polymer matrix during the curing process due to relatively high density. At a low filler loading, the upper part of the composite is barely neat epoxy, while the lower part is comprised of sunk filler particles. Submerged particles function as thermal reservoirs that impede thermal transport in the through-plane direction of the composites [19]. When increasing its content, the filler is piled up from the bottom of the composite. Moreover, when the content of BN in 3 g of epoxy reaches 1 g, full packing of fillers in the epoxy occurs. In this state, uniform dispersion of fillers inside the polymer matrix is achieved, resulting in more efficient thermal transport, reaching the thermal percolation threshold [8]. Meanwhile, there is no significant difference in thermal diffusivity between composites filled with raw a-BN and hybrid fillers before full packing. In this state, interconnecting hybrid fillers through coalescence of SAC305 cannot contribute effectively to heat transfer. At an a-BN loading of 1 g in 3 g epoxy, higher thermal diffusivity is measured in hybrid fillers with a higher ratio of SAC305. Consequently, above full filler packing, additional SAC305 particles can function as interconnecting points between a-BN particles after the coalescence behavior, promoting the formation of an elaborate percolative 3D network [20]. The thermal diffusivity of neat epoxy and composites separately filled with SAC305 and a-BN were compared to confirm the influence of coalescence behavior on composites with hybrid fillers. First, when SAC305 alone was added into 3 g of epoxy (0.165 g, 0.375 g, 0.645 g, and 1 g), thermal diffusivity increased only slightly (Table S1) compared with that of neat epoxy. Then, an equivalent amount of a-BN alone was added into 3 g of epoxy; the thermal diffusivity of the composite filled with 1 g of a-BN reached 0.602 mm^2^/s. The thermal diffusivity enhancement ratio of neat epoxy to SAC305 (1 g)/epoxy (3 g) and a-BN (1 g)/epoxy (3 g) to epoxy (3 g) composite filled with 2 g of a-BN/SAC305 (1:1 ratio) are shown in Figure S2. While the former is 10%, the latter increases to 59% with the same amount of SAC305 added. This enhancement confirms that the high-thermal diffusivity of hybrid filler composite is more induced by the construction of 3D interconnected networks via coalescence of SAC305 than the intrinsic thermal property of SAC305 itself.

Figure 2b,c presents the density and heat capacity of the composites. Because SAC305 has a higher density (7.38 g/cm^3^) than a-BN (2.1 g/cm^3^), the density of the composites increases when the content of the hybrid fillers increases, and this increase stands out for higher SAC305 ratio. Contrary to the tendency of density, the heat capacity of the composites decreases with increasing hybrid fillers due to the low heat capacity of SAC305 (metal, 0.232 J/gK), compared to a-BN (ceramic, 0.79 J/gK) and epoxy (polymer, 1.11 J/gK). For epoxy composite (3 g) filled with a 1:1 ratio hybrid filler (2 g), the heat capacity decreases to 0.6 J/gK, which is 54% that of neat epoxy. The thermal conductivity of the composites was calculated by multiplying measured thermal diffusivity by the specific heat capacity and density of the composites [21]. Based on the a-BN content (1 g) on 3 g of epoxy, a 1:0.5 ratio of hybrid filler composite reached the maximum of 0.95 W/mK (compared to the others), while the highest thermal diffusivity was achieved in a 1:1 ratio. This result is attributed to the low heat capacity of SAC305. Materials for TIM applications need to propagate heat inside the device quickly to the outside; simultaneously, the temperature of the TIM itself should not heat up easily. Therefore, additional SAC305 could form additional contact points between a-BN particles after coalescence, producing fast heat propagation. However, excessive SAC305 results in a lower specific heat capacity of the composites, which is detrimental to using it as a TIM [22]. Consequently, a 1:0.5 ratio of hybrid filler composite reaches the optimal point in thermal conductivity and could be selected as the most suitable for TIM applications. It is also worth noting that enhanced thermal conductivity of the composites was measured when cured at mp of SAC305 compared to below the mp, indicating a beneficial effect of coalescence behavior on the heat dissipation (Figure S3).

**Figure 3:**
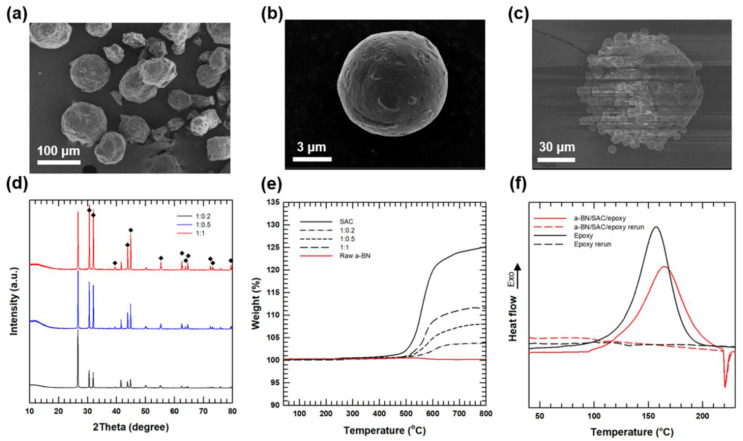
Field-emission scanning electron microscope (FE-SEM) images of (**a**) aggregated boron nitride (a-BN), (**b**) SAC305, and (**c**) 1:1 wt ratio of a-BN/SAC305 hybrid filler particle. (**d**) XRD pattern of the hybrid fillers with various wt ratio. (**e**) Thermogravimetric analysis (TGA) curves for raw a-BN, SAC305, and hybrid fillers with various wt ratios under air atmospheres. (**f**) In-situ differential scanning calorimetry (DSC) curves of neat epoxy resin and epoxy resin mixed with hybrid filler. The dashed lines show the curves for second heating scan.

Figure 3a–c shows the FE-SEM images of a-BN particles, SAC305, and SAC305-attached a-BN hybrid filler. As depicted in Figure 3a,b, a-BN and SAC305 have spherical morphology with average particle sizes of 70 and 6 μm, respectively. Figure 3c shows the morphology of the a-BN/SAC305 hybrid filler with a 1:1 wt ratio. During the stirring and vacuum filtration process, SAC305 particles could be easily attached to the a-BN surface by van der Waals attraction force between metal and ceramic [23,24]. Figure 3d presents the XRD pattern of hybrid fillers with different wt ratios. The intensity of the peaks from SAC305 (marked with diamond symbol) increases with input ratio when normalized by the (002) peak of BN at an angle 2θ of 26.7°.Quantitative analysis was also conducted by measuring TGA in air, which is the curing process atmosphere used in this study (Figure 3e). There is no weight loss or gain for pristine a-BN, demonstrating thermal stability. For SAC305, the weight gain occurs near 400 °C, and the maximum weight is approximately 125% of its initial weight at 800 °C, corresponding to thermal oxidation [25]. For hybrid fillers, weight gain by thermal oxidation is increased with an increase in the ratio of SAC305; moreover, the weight gain percent at 800 °C of each sample corresponds well with the input ratio of SAC305. 

Figure 3f shows the in-situ DSC curves of the epoxy resin and epoxy resin mixed with hybrid filler to monitor the curing and coalescence behavior. For neat epoxy resin, the maximum exothermic peak appears at 157.2 °C, which is associated with the curing process. This peak shifts to higher temperatures for epoxy resin mixed with a hybrid filler, likely due to restricted chain mobility caused by filler between the polymer backbone. The curing process occurs at a higher temperature because of the impeded crosslinking of the polymer chains. Moreover, the peak intensity decreases compared to neat epoxy because less heat is generated per unit mass due to the additional filler [26]. Coalescence behavior is confirmed by the endothermic peak at 220 °C, corresponding to the melting of SAC305 particles. Both resins lack exothermic peaks after reruns because they already cured during the first measurement.

**Figure 4:**
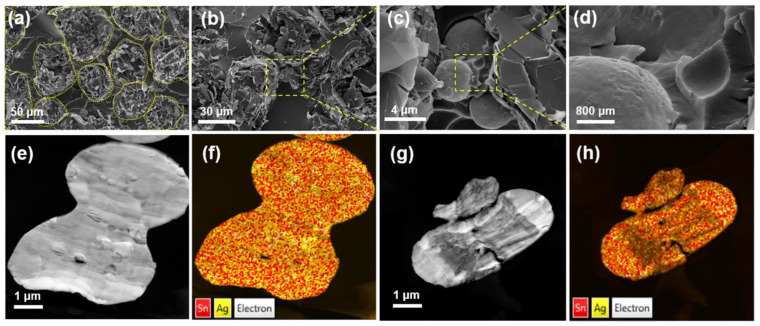
(**a**) Cross-sectional FE-SEM images of epoxy composites filled with 40% filler fraction of hybrid filler (1:1 wt ratio), and (**b**–**d**) their high magnification images. The yellow part in (**a**–**c**) shows the bridging morphologies. (**e**,**g**) Cross-sectional FE-TEM images of epoxy composites filled with 40% filler fraction of hybrid filler (1:1 wt ratio), and (**f**,**h**) their EDS atomic analysis.

The coalescence behavior of SAC305 was further characterized by observing the fractured surface of the composites using FE-SEM and FE-TEM. Figure 4a illustrates the FE-SEM image of a-BN particles tightly packed inside the matrix. The interfacial phonon scattering between epoxy and a-BN impedes thermal conduction in the composites [27]. However, bridging fillers with SAC305 could solve this problem by constructing thermal transport paths to reduce thermal resistance. The bridging morphologies are depicted in magnified FE-SEM images (Figure 4b–d). Spherical SAC305 particles are coalesced to each other, and this was further confirmed by FE-TEM of the composites fractured surface. Figure 4e,g shows coalesced SAC305 particles; their EDS mapping images are shown in Figure 4f,h, respectively. No other visible image was detected except SAC305 in the composites, because of the strong signal from metal particles, compared to the ceramic filler and polymer matrix. These well-connected filler images also explain the decision of 10 min of curing time at 220 °C in this study. When we set the curing time at 220 °C for 30 min, too much melting occurred and flowed to the bottom of the composite, coalescing into huge particles when cooled (Figure S4).

## 4. Conclusions

We fabricated a hybrid filler by attaching SAC305 on an a-BN surface using simple stirring with solvent, followed by the vacuum filtration method. It was then incorporated into epoxy resin, and a 3D thermal path was formed during the curing process via the coalescence behavior of SAC305. The adjacent a-BN particles were connected to each other inside the epoxy matrix, which exhibited enhanced thermal conductivity of 0.95 W/mK in 1:0.5 hybrid filler composite with 1 g of a-BN on 3 g epoxy. In contrast, that of raw a-BN was 0.82 W/mK with an equivalent amount of a-BN. The 3D filler network built with SAC305 as a bridging material interconnecting a-BN particles enabled efficient heat transport because the fillers reduced the thermal resistance between the filler and matrix. Moreover, the morphologies of the 3D filler network were further confirmed by FE-SEM and FE-TEM measurements of the fractured composite surface. These results provide evidence of the exclusive effect of enhancing thermal conductivity by constructing a 3D thermal path using SAC305, which has low thermal conductivity, as bridging material.

## Figures and Tables

**Table 1:** Experimental condition for preparation of composites with various ratios of the hybrid fillers.

Filler (wt%)	Amount of Filler (g)	a-BN (g)	SAC305 (g)	a-BN:SAC wt Ratio
10%	0.33	0.165	0.165	1:1
20%	0.75	0.375	0.375	1:1
30%	1.29	0.645	0.645	1:1
40%	2	1	1	1:1
8%	0.25	0.165	0.083	1:0.5
16%	0.56	0.375	0.188	1:0.5
24%	0.97	0.645	0.323	1:0.5
33%	1.50	1	0.500	1:0.5
6%	0.20	0.165	0.033	1:0.2
13%	0.45	0.375	0.075	1:0.2
21%	0.77	0.645	0.129	1:0.2
29%	1.20	1	0.200	1:0.2

## Supplementary Materials

The following are available online at https://www.mdpi.com/2073-4360/12/9/1954/s1, Figure S1: Cross-sectional FE-SEM images of epoxy composites filled with (a) 10%, (b) 20%, (c) 30%, and (d) 40% filler fraction of hybrid filler (1:1 wt ratio), Table S1: Experimental condition for preparation of composites with various fillers, and their measured thermal diffusivities, Figure S2: Schematic of the composites of #1, #5, #9, and #13 in Table S1, and the thermal diffusivity enhancement ratio of #1 to #5 and #9 to #13, Figure S3: Thermal conductivity of the composites filled with 40% filler fraction of hybrid filler (1:1 wt ratio), 33% filler fraction of hybrid filler (1:0.5 wt ratio), and 29% filler fraction (1:0.2 wt ratio) of hybrid filler cured at 180 °C and 220 °C, Figure S4: FE-SEM image of over coalesced SAC305 particle in fractured surface of the composite when curing temperature set to 220 °C for 30 min.
