# Supplementary material for: 3D Interconnected Boron Nitride Networks in Epoxy Composites via Coalescence Behavior of SAC305 Solder Alloy as a Bridging Material for Enhanced Thermal Conductivity
Source: Polymers (Basel). 2020 Aug 28;12(9):1954. doi: 10.3390/polym12091954 (PMC7564005; doi:10.3390/polym12091954)
Supplement: Supplementary file 1 [file polymers-12-01954-s001.pdf]

Supplementary material

3D interconnected boron nitride networks in epoxy composites via coalescence behavior of SAC305 solder alloy as a bridging material for enhanced thermal conductivity

Youjin Kim and Jooheon Kim*

School of Chemical Engineering and Materials Science, Chung-Ang University,

Seoul 156-756, Republic of Korea

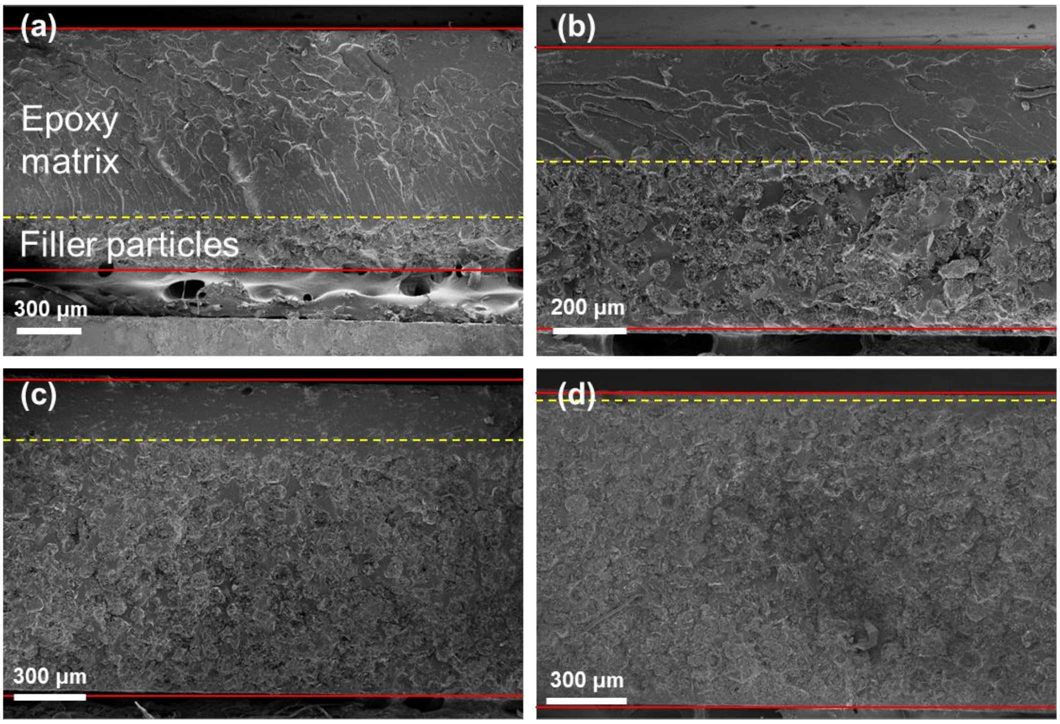

Figure S1. Cross-sectional FE-SEM images of epoxy composites filled with (a) 10%, (b) 20%, (c) 30%, and (d) 40% filler fraction of hybrid filler (1:1 wt ratio).

Table S1. Experimental condition for preparation of composites with various fillers, and their measured thermal diffusivities.

Experimental #	Epoxy (g)	a-BN (g)	SAC305 (g)	Thermal diffusivity (mm^2/s)
1	3	0	0	0.146
2	3	0	0.165	0.15
3	3	0	0.375	0.156
4	3	0	0.645	0.152
5	3	0	1	0.161
6	3	0.165	0	0.166
7	3	0.375	0	0.214
8	3	0.645	0	0.308
9	3	1	0	0.602
10	3	0.165	0.165	0.236
11	3	0.375	0.375	0.264
12	3	0.645	0.645	0.444
13	3	1	1	0.957

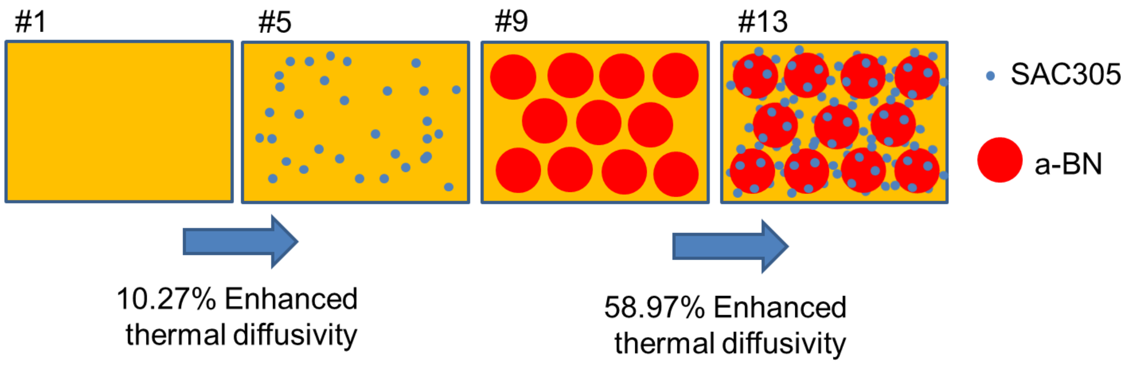

Figure S2. Schematic of the composites of #1, #5, #9, and #13 in Table S1, and the thermal diffusivity enhancement ratio of #1 to #5 and #9 to #13.

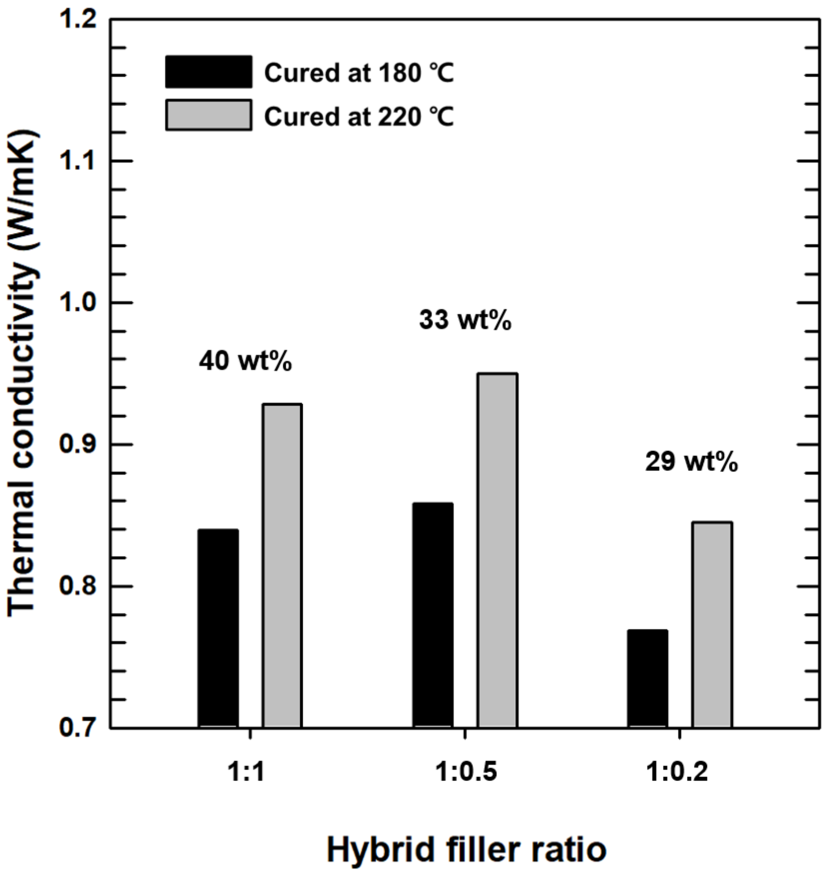

Figure S3. Thermal conductivity of the composites filled with 40% filler fraction of hybrid filler (1:1 wt ratio), 33% filler fraction of hybrid filler (1:0.5 wt ratio), and 29% filler fraction (1:0.2 wt ratio) of hybrid filler cured at 180 ℃ and 220 ℃.

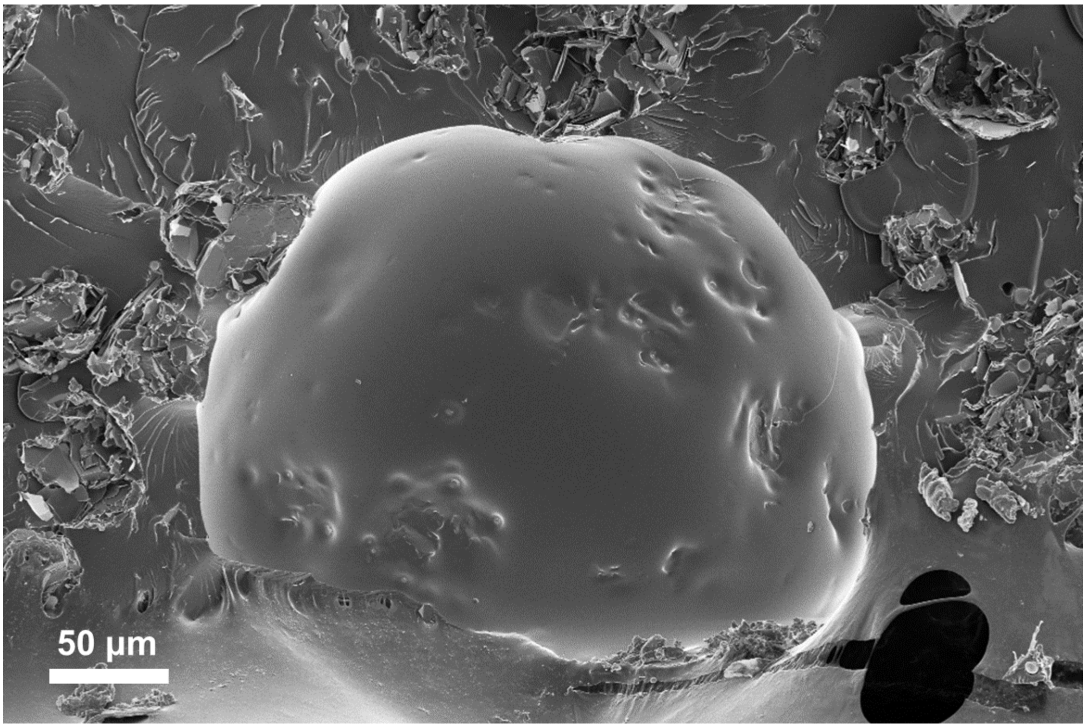

Figure S4. FE-SEM image of over coalesced SAC305 particle in fractured surface of the composite when curing temperature set to 220 ℃ for 30 min.
